# Prostatic artery embolization with polyethylene glycol microspheres: evaluation in a canine spontaneous benign prostatic hyperplasia model

**DOI:** 10.1186/s42155-020-00130-5

**Published:** 2020-09-06

**Authors:** Vanesa Lucas Cava, Francisco Miguel Sánchez Margallo, Claudia Báez Díaz, Luis Dávila Gómez, Juan Rafael Lima Rodríguez, Fei Sun

**Affiliations:** 1 Endoluminal Therapy and Diagnosis Unit, Jesús Usón Minimally Invasive Surgery Centre, Carretera N-521, km 41.8, 10071 Cáceres, Spain; 2 Scientific Director, Jesús Usón Minimally Invasive Surgery Centre, Cáceres, Spain; 3 Animal Housing Service, Jesús Usón Minimally Invasive Surgery Centre, Cáceres, Spain; 4 Anaesthesiology Unit, Jesús Usón Minimally Invasive Surgery Centre, Cáceres, Spain

**Keywords:** Benign prostatic hyperplasia, Canine model, Microspheres, Prostatic artery embolization

## Abstract

**Background:**

Prostatic artery embolization (PAE) is a minimally invasive technique for the management of symptomatic benign prostatic hyperplasia (BPH) relieving the lower urinary tract symptoms in patients. Various embolic agents have been tested in animal models and subsequently used in human patients. The purpose of this study was to evaluate the technical feasibility, effectiveness, and safety of PAE with polyethylene glycol microspheres in a canine spontaneous BPH model.

**Results:**

Five adult male Beagle dogs (4.78 ± 1.11 years) were diagnosed by tranrectal ultrasonography of spontaneous BPH (prostate volume > 18 ml) and underwent PAE with polyethylene glycol microspheres (400 ± 75 μm). PAE procedures were performed successfully in all dogs. After PAE, all dogs were inspected for potential procedure-related complications during 1 month of follow-up. No major complications were observed any animal. Follow-up angiography was performed in each animal at 1 month of follow-up. Recanalization was demonstrated in all the embolized prostatic arteries or main branches at the end of the study. Magnetic Resonance Imaging (MRI) evaluations were performed immediately before PAE as baseline data, and 1 week, 2 weeks and 1 month after PAE. MRI study showed that the prostate shrank substantially with ischemic necrosis in each dog. There was a significant reduction in the mean prostate volume at 2 weeks and 1 month compared with the baseline data, from 19.95 ± 1.89 mL to 13.14 ± 2.33 and 9.35 ± 2.69 mL (*p* < 0.001), respectively. Histopathological study was conducted after 1-month follow-up angiography and confirmed the therapeutic responses with diffuse glandular atrophy and interstitial fibrosis.

**Conclusions:**

The findings of the present study support that PAE with the use of polyethylene glycol microspheres is a safe and feasible procedure that may induce a significant shrinkage of prostate due to the local ischemia and secondary glandular atrophy. Early recanalization of target arteries remains to be further addressed in both laboratory investigation and clinical practice.

## Background

During the past decade, prostatic artery embolization (PAE) for the treatment of symptomatic benign prostatic hyperplasia (BPH) has been successfully translated from bench research to clinical practice as a promising alternative to prostate surgery or medical therapies (Sun et al. 2008; McWilliams et al. 2019). A recent official approval of PAE for the routine treatment of clinical BPH by National Institute of Health and Care Excellence (NICE) indicated that PAE is no longer a clinical experimental procedure in the UK and some other countries that follow the NICE guidance (Excellence 2018; Hacking 2018). While PAE has gained in popularity worldwide, there is increasing interest in development of new devices, such as embolic agents, and in modifications of PAE techniques. To test new devices and address technical modifications a reliable animal model of BPH is essential. Animal models used in previous preclinical studies in evaluation of PAE techniques included large-white pigs, hormone-induced BPH models in beagles and intact old dogs, each model of which has its inherent drawbacks and clinical limitations (Sun et al. 2008; Sun et al. 2011; Brook et al. 2013). More recently, Sun et al. (2017) introduced the use of canine spontaneous BPH model and its selection criteria of prostate volume (PV) larger than 18 mL, due to the presence of histological changes of hyperplasia in canine prostates with that size. To our best knowledge, this is the first animal experimental report using canine spontaneous BPH model to test polyethylene glycol-based microsphere that has a potential in clinical practice in PAE. Polyethylene glycol microsphere is a biocompatible, compressible, non-drug-loadable, non-absorbable embolic agent, which is designed for bland embolization of hypervascular tumors. The purpose of this study was to evaluate the technical feasibility, clinical safety and therapeutic effects in a novel canine spontaneous BPH model.

## Methods

### Animals

The animal experiment protocol was approved by Institutional Ethic Committee of Animal Experimentation. Screening for spontaneous BPH in dogs by transrectal ultrasound (TRUS) was performed 1–2 weeks before PAE procedures. As standard inclusion criteria of spontaneous BPH in dogs was used prostate size larger than 18 g or mL suggested by Sun et al. (2017). Five adult male Beagle dogs (mean body weight 17.30 ± 2.44 kg) with PV larger than 18 mL (mean PV 19.95 ± 1.88 mL) were enrolled in this study. The mean age of beagles was 4.78 ± 1.11 years with a range of 3.5–6.4 years.

### PAE procedure and angiography follow-up

**Fig. 1:**
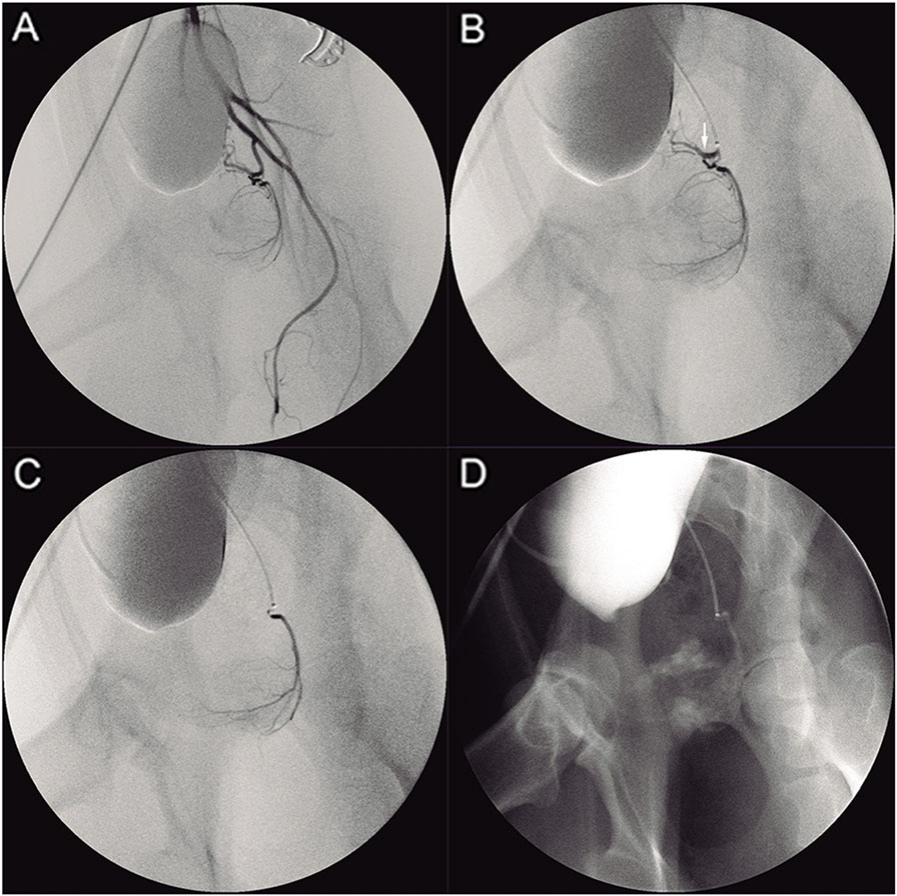
PAE procedure in a dog with spontaneous BPH. Left internal iliac arteriogram (**a**) shows the prostatic artery arises from the anterior division of the internal iliac artery. **b** Shows the microcatheter in the left prostatic artery. The white arrow indicates the cranial branch (caudal vesical artery) of the prostatic artery. **c** Shows the tip of microcatheter in the prostatic branch, where microspheres are injected. **d** Shows radiogram immediated after PAE on the left side, indicating the mixture of microspheres with contrast medium injected into part of the left lobe

After fasting for 24 h, each dog was anesthetized with propofol (Diprivan, AstraZeneca S.p.A. Caponago, Milán, Italia) 3 mg/kg intravenously, intubated endotracheally and maintained with inhaled concentrations of 3.3–3.6% sevoflurane (Sevorane; Abbott Laboratories, Madrid, Spain) in an anesthesia system and mechanical ventilator (Leon Plus; Heinen and Löwenstein GmbH, Bad Ems, Germany). The dogs were placed in supine position and under sterile conditions, the right femoral artery access was established by Seldinger technique with a 4 Fr introducer sheath (Radifocus, Terumo Medical, Somerset, NJ, USA). Under fluoroscopy, a 4 Fr angiographic catheter (Simmons Sidewinder I, Terumo Medical, Somerset, NJ, USA) was placed into the main trunk of the left internal iliac artery or its anterior branch. A 2.4 Fr microcatheter (Progreat, Terumo Medical, Somerset, NJ, USA) with a 0.016″ microwire (GT, Terumo Medical, Somerset, NJ, USA) was inserted coaxially through the angiographic catheter and positioned into the prostatic artery. Superselective angiography in the left prostatic artery was performed with manual injection of diluted contrast medium (Omnipaque 240 ng I /mL, GE Healthcare, Madrid, Spain). Polyethylene glycol microspheres (2 mL/vial) (HydroPearl™ 400 ± 75 μm, MicroVention, Tustin, CA, USA) was then diluted with the same amount of 100% contrast medium as the phosphate-buffered saline preloaded in the embolic syringe. An ideal target site to selectively place the microcatheter tip before embolization was at the part of prostatic artery distal to the origin of the caudal vesicle artery (Fig. 1). Alternatively, the main trunk of the prostatic artery, proximal to its branching into the caudal vesical artery was acceptable target site if angiography with the microcatheter confirmed that contrast medium predominantly filled the prostatic vasculature. Once the microcatheter was appropriately positioned, microspheres were slowly injected under guidance of fluoroscopy or road-mapping. Embolization was terminated when complete stasis inside the prostate vasculature was obtained or reflux of the mixture of embolic agent and contrast medium toward the caudal vesical artery, middle rectal artery or internal pudendal artery was observed. The amount of microspheres used was recorded, and control angiography in the internal iliac artery was performed to ensure the complete stasis was achieved. PAE on the right site was performed using the same protocol above. At completion of the intervention, the procedure times, radiation times, and radiation doses were documented.

After PAE, the animals were recovered from general anesthesia and returned to animal housing. Each animal was routinely administered with analgesics and antibiotics for 3 to 5 days, respectively after PAE. Follow-up angiography was performed at 1 month after PAE immediately before euthanasia of the animals.

### Postprocedural clinical observation

Clinical observation after PAE was conducted by research veterinarians. Each dog was checked twice a day for 1 week and then once daily until 1 month. The dogs’ general conditions such as body temperature, appetite and vitality were recorded. The potential complications associated with PAE due to nontarget embolization, such as acute urinary retention, sign of peritonitis, penile necrosis, hematuria, blood in the stool, skin or muscle ischemic necrosis and hematoma at the puncture site were closely inspected. Clinical management of the complications was conducted when necessary.

### MRI evaluation

Magnetic Resonance Imaging (MRI) examinations were performed in all dogs with a 1.5 T system (Intera; Philips Medical Systems) before PAE as baseline data and 1 week, 2 weeks and 1 month after PAE to assess the morphologic responses and measure the PV. Under general anesthesia, the animals were placed in a supine position with a SENSE-Flex-M coil (Philips Medical Systems) around the lower abdomen for image acquisition. A typical MRI examination included axial T1-weighted turbo spin-echo and T2-weighted turbo spin-echo images with a field of view of 14 × 14 cm and a 3 mm section thickness in a 232 × 232 matrix. An additional contrast enhanced T1-weighted imaging was also obtained by intravenous administration of a bolus of 0.1 mmol/kg gadopentetate dimeglumine (Magnevist®; Shering AG). All the imaging data were collected for subsequent imaging analysis by two investigators (F.S and V.L.C).

### Necropsy and Histopathologic study

After 1-month follow-up of MRI and angiography, all animals were euthanized under general anesthesia with overdose of potassium chloride and subjected to necropsy. The prostate and surrounding structures in the pelvis, including the urinary bladder, rectum, vas deferens, and urethra were carefully inspected for pathological changes. The prostates were harvested and fixed with 10% neutral buffered formalin, and subsequently the specimens were sectioned axially into five blocks for macroscopic study. Tissues were dehydrated in a graded series of ethanol and embedded in paraffin. Sections were cut into 5 μm thickness and stained with hematoxylin and eosin for histological study.

### Statistical analysis

The statistical analysis was conducted with statistical software package SPSS version 24. The descriptive analysis was performed in all variables and expressed as means ± standard deviation. The normality study was using the Shapiro-Wilk test. The paired sample t-test was used to compare the mean PV at different time points. *P* value < 0.05 was considered to indicate a statistically significant difference.

## Results

### PAE procedure

Technical success, which was defined as complete stasis confirmed with control angiography immediately after PAE in bilateral embolization, was obtained in all cases. The mean procedure times, mean radiation times and mean radiation doses were 97.60 ± 22.23 min, 32.28 ± 8.66 min and 113.64 ± 35.05 mGy, respectively. The mean dosage of injected microspheres was 1.19/2 mL of each vial in the whole prostate.

### Clinical observation

No major complications after PAE, such as untoward embolization-induced ischemia of the urinary bladder, rectum or the glans, and severe urinary sepsis, were observed in the present study. One dog had decreased appetite at 6 h after PAE. The 2nd dog was observed with a hematoma at the femoral arterial puncture site on Day 2. Both animals underwent routine administration of antibiotics and analgesics without specific clinical treatment. The 3rd dog suffered from fever (40.3 °C) with lower appetite on Day 10 after PAE. The body temperature became normal 5 days later after administration of antibiotics.

### MRI evaluation

**Fig. 2:**
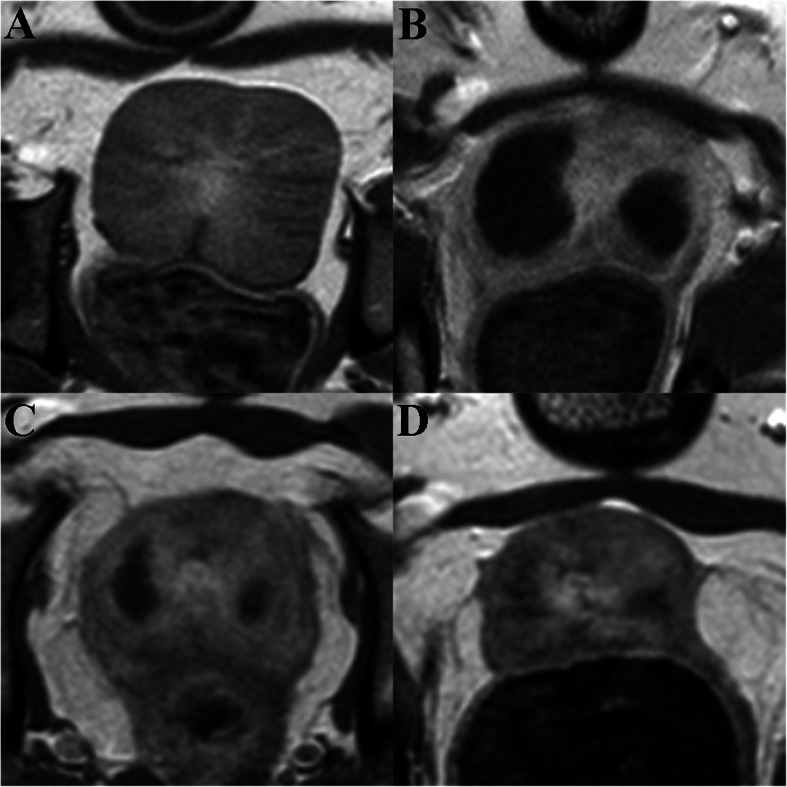
Contrast enhanced axial T1-weighted. MR images show​ the prostate immediately before PAE (**a**), 1 week (**b**), 2 weeks (**c**), and 1 month (**d**) after PAE. The infarct lesions lacking of enhancement gradually decrease in size with time and the prostate shrinks substantially at 1-month follow-up (**d**)

Prostate size evaluated by MRI showed a significant decrease (*p* < 0.001) at 2 weeks and 1 month after PAE but not at 1 week compared with baseline data (Table 1 and Fig. 2). Early morphologic responses to PAE were detected in all prostates at 1 week after PAE, as non-enhanced areas surrounded by hyperintense signal on contrast-enhanced T1-weighted images corresponding to the presence of ischemia infarction and inflammation, respectively. Cavitary necrosis was found in 3 out 5 dogs.

**Table 1:** MRI measurements: prostate volume (mL) at different times of follow-up

	Baseline	1 week	2 weeks	1 month
**Dog 1**	20.77	16.83 (−18.97%)	13.17 (−36.59%)	12.70 (−38.85%)
**Dog 2**	18.78	22.81 (21.46%)	13.20 (−29.71%)	6.97 (−62.89%)
**Dog 3**	18.90	18.21 (−3.65%)	10.75 (− 43.12)	6.51 (−65.56%)
**Dog 4**	18.41	13.04 (−29.17%)	11.70 (−36.45%)	9.19 (−50.08%)
**Dog 5**	22.90	19.80 (−13.54%)	16.87 (− 26.33%)	11.36 (−50.39%)
**Mean ± DS**	19.95 ± 1.88	18.14 ± 3.62 (− 9.07%)	13.14 ± 2.33* (− 34.14%)	9.35 ± 2.69* (− 53.13%)

*p* < 0.001*

Values in parentheses are percentage of prostate volume changes compared with baseline data

### Follow-up angiography

**Fig. 3:**
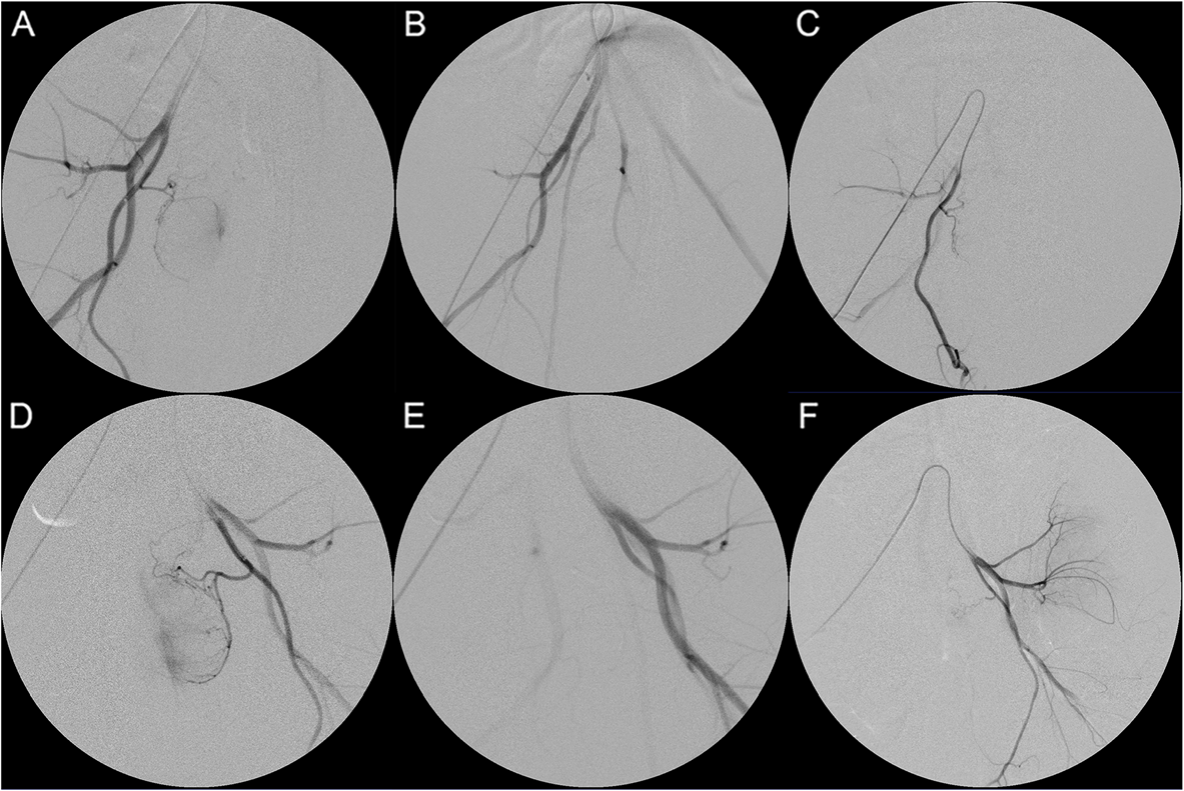
Selective arteriography before (**a**) and (**d**), immediately after (**b**) and (**e**), and 1 month after PAE (**c**) and (**f**). The images in the upper row are the right prostatic arteriograms; those in the lower row are the left prostatic arteriograms in the same animal. Note occlusion of the prostatic artery in (**b**) and (**e**) and recanalization in (**c**) and (**f**). The right prostatic artery shows total recanalization (**c**) and the left one shows the proximal obstruction with distal fine new vessels (**f**)

Follow-up angiography was performed in each animal at the end of the study. All animals showed patency of the prostatic arterial trunk or its major branches on both sides, including total recanalization in 3 prostatic arteries and partial recanalization in 7 prostatic arteries (Fig. 3).

### Necropsy and Histopathologic study

**Fig. 4:**
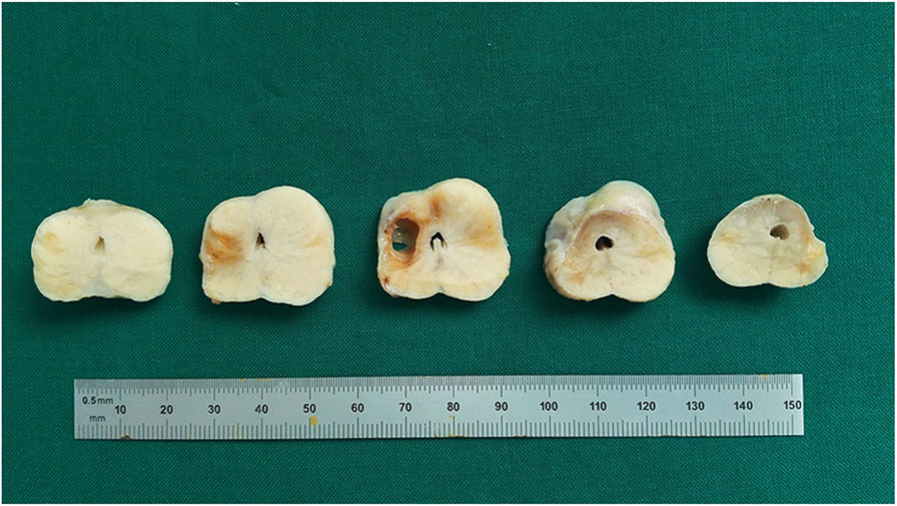
Macroscopic picture of the prostate shows a prostate specimen sectioned in 5 mm thickness with multiple focal hemorrhagic necrosis and cavity formation

**Fig. 5:**
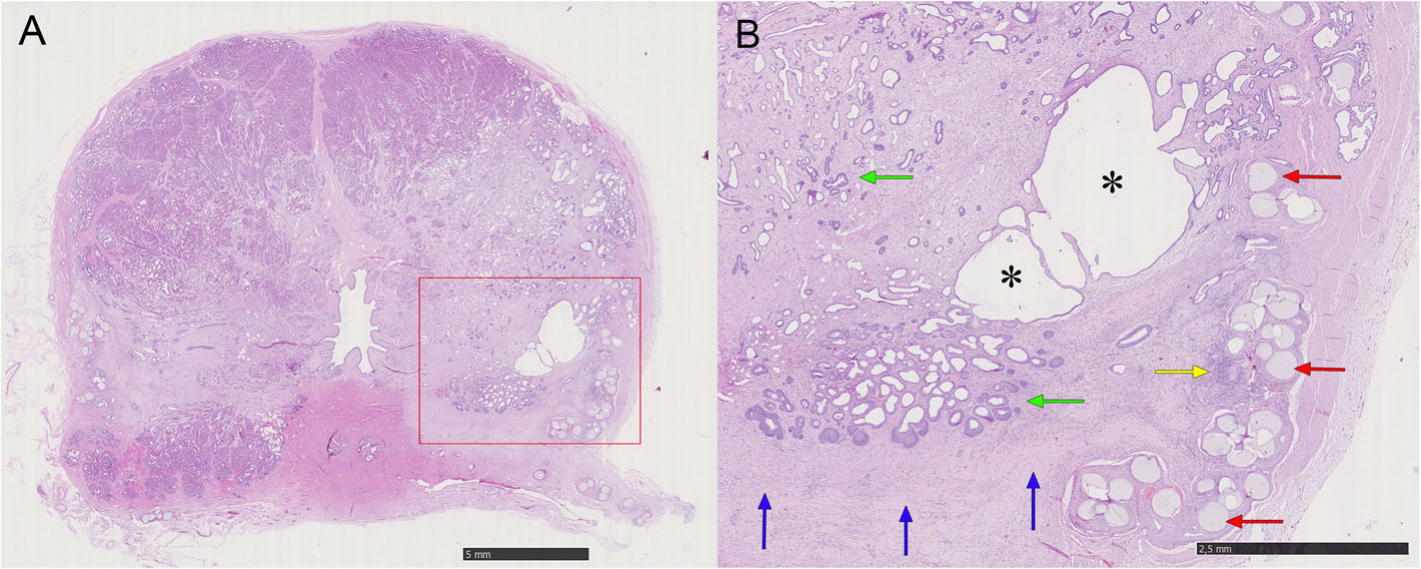
Microscopic pictures (Hematoxylin & Eosin staining) in the same dog as showed in Fig. 2. **a** Shows a cavity formation in the left lobe (without magnification). **b** Shows the low-powered view of the square portion in (**a**). Note the microspheres in the occluded arterioles (red arrows), cavitary formation (asterisks), atrophic residual gland (green arrows), inflammatory cell infiltration (yellow arrow), and diffuse interstitial fibrosis (blue arrows)

In the necropsy, the prostate shrank substantially in all dogs. No abnormalities were found in surrounding organs, including the urinary bladder, rectum and vas deferens. In macroscopic examination, multiple focal areas of hemorrhagic necrosis were observed in prostates and some specimens showed cavity formation (Fig. 4). In microscopic study, the diffuse glandular atrophy, interstitial fibrosis, inflammatory cell infiltration and non-tissue intraprostatic areas of cavity formation were identified (Fig. 5). The injected intra-arterial microspheres were also observed at the periphery of the glands.

## Discussion

Aside from nonhuman primate, the dog is the only nonhuman specie in which spontaneous BPH occurs physiologically. As like in humans, BPH in dogs is an age-related disease which causes a hyperplastic prostate growth (Sun et al. 2017). In a pathological study in 41 intact beagles (1–10 years), DeKlerk et al. (1979) identified two types of spontaneous BPH, glandular hyperplasia and cystic hyperplasia (complex form of hyperplasia). The canine BPH may develop as early as 2–3 years of age as a glandular hyperplasia characterized as an adenomatous disease, which later becomes cystic hyperplasia with hyperplastic epithelium combined with formation of large cysts and increase in the stroma amount (Sun et al. 2017; DeKlerk et al. 1979; Palmieri et al. 2019). Thus, just the cystic or complex form of hyperplasia in dogs shares more similarities to human BPH in pathology and therefore, it would be an ideal canine model in evaluation of PAE technique and associated devices. In addition, in this report was observed that all prostates that weighted more than 18 g had pathological evidence of BPH; whereas all except one that weighted less than 12.8 g were histologically normal. Accordingly, Sun et al. (2017) suggested that the gravimetric criterion of prostate weight larger than 18 g in adult beagles can be used as a standard in screening for spontaneous BPH model in preclinical studies to test new devices in PAE procedures.

Hormone-induced BPH canine models are currently most commonly used in evaluation PAE techniques and interventional devices (Sun et al. 2011; Jeon et al. 2009). According to Sun et al. (2017), hormone-induced BPH is a reliable and reproducible model that can be established in young surgical castrated or intact dogs with common hormonal treatment regimens based on combination of androgens and estrogens for 3 months or longer. One striking feature of this model is its large size of the prostate and large diameter of the prostatic arteries. A report demonstrated that after 3-month hormonal therapy, the prostate volume increased up to 572% (Sun et al. 2011). Another advantage of this model is the use of young beagles (1–2 years), which is of much more commercial availability compared with the older beagles with spontaneous BPH.

However, hormone-induced BPH model in evaluation of PAE techniques has several disadvantages. In pathology, all hormone-induced BPH models represent the glandular hyperplasia rather than the complex form of hyperplasia with mild to moderate increase in the stroma that is observed in old dogs with spontaneous BPH (DeKlerk et al. 1979). The lack of stroma tissue in the hormone-induced model may explain the highly sensitive responses to PAE, such as massive intraprostatic infarction and secondary cavity formation (Sun et al. 2017), in comparison with findings in human patients, where cavity formation rarely occurred after PAE (Frenk et al. 2014). Moreover, hormone-induced BPH models are not suitable for chronic studies with long-term evaluation after PAE because hormonal therapy should last in whole duration of the study; otherwise, the cessation of administration of the hormones may lead to atrophy of the prostate (Jeon et al. 2009). Due to the prostate gland grows rapidly when it is exposed to high serum level of exogenous androgens and estrogen, if the recanalization of the prostatic arteries occurs or the collateral circulation is established after PAE, the residual glandular tissue has a tendency to rapidly regenerate and grow in size (Jeon et al. 2009; Zhang et al. 2020). Obviously, this situation would occur neither in dogs with spontaneous BPH nor in human patients in clinical practice. In addition, hormone-induced BPH cannot be used in evaluation of sexual function following PAE, which can be conducted in spontaneous BPH model.

It is worth noting that canine models of both spontaneous BPH and hormone-induced BPH have their inherent limitations in evaluation of PAE techniques. Unlike in human patients, the canine hyperplastic prostate expands outwardly in all directions, commonly observed clinical signs involve those of rectal obstruction rather than lower urinary tract symptoms (LUTS). Furthermore, prostate specific antigen (PSA), an important biochemical marker in clinical practice in human prostatic disorders, is not detected in canine blood or seminal fluid (Sun et al. 2017). Therefore, evaluation of LUTS, urodynamic study, and test of PSA are not applicable in preclinical studies of PAE techniques in dogs. Alternatively, the only likely parameter to evaluate the therapeutic effects of PAE is the prostate volume change detected by imaging technologies, MRI or ultrasonography.

In the present study, substantial decrease in PV was detected early at 2 weeks, and the prostates further shrank at 1 month after PAE. Compared with the baseline data, the PV reduction was about 34% at 2 weeks and 53% at 1 month, respectively, supporting the therapeutic effects of PAE with polyethylene glycol microspheres. However, no significant change in the PV was noticed at 1 week, which may be explained by the MRI findings detected of intraprostatic infarction and edema of surrounding tissue; with time, as the edema is absorbed and organization or fibrosis occurs in the infarcted tissue, the prostate will shrink (Sun et al. 2016). This mechanism of shrinkage of prostate after PAE was also supported by the pathological findings in our present study. Cavitary necrosis is a common pathological reaction to PAE in dogs, which has also been reported in other studies (Sun et al. 2011; Jeon et al. 2009). This finding is caused by ischemia in glandular prostatic tissue whose area of necrosis gradually sloughts forming an intraprostatic cavity. The present study showed cavitary necrosis in 3 out of 5 dogs; whereas in a previous study on PAE in hormone-induced model, cavitary necrosis was observed in all 7 dogs (Sun et al. 2011). The inconsistent findings may be attributed to the different pathological features between the both BPH models. In PAE procedures of present study, technical success with bilateral embolization was achieved in all cases, highlighting the technical feasibility of the embolic agent as well as reliability of the animal model. In addition, no major complications related to PAE procedures were observed in any animal, indicating the technical safety of PAE by the use of the microspheres.

Interestingly, we observed that the prostatic artery or its main branches reopened in all dogs at 1-month follow-up of angiography. The recanalization varied in morphology, including the wide open lumen as total recanalization and the narrowed lumen or proximal obstruction together with distal fine anastomosis as partial recanalization. It is not surprising that recanalization occurs after arterial embolization with non-absorbable embolic agents. In a report of embolization in porcine renal model, four different embolic agents, spherical and non-spherical particles, were used to embolize the upper pole of the kidney. At 28 days after embolization, the follow-up angiographies showed partial recanalization with non-spherical particles while all but one of the arteries embolized with spherical particles were recanalized (Siskin et al. 2003). Similarly, Bilbao et al. (2008) tested four spherical embolic agents in pig kidney. The follow-up angiographies after 4 weeks postembolization showed variable degree of recanalization with three out of four embolic agents. The underlying recanalization mechanism has been suggested by the inflammatory process related to the injury in the embolized arterial wall that promotes the resorption of thrombus, angiogenesis and capillary regrowth, so as the particles exclusion from the occluded vessel wall (Laurent et al. 2009). Besides, capillary development is induced by releasing of the angiogenic factors under hypoxia conditions associated to embolization (Keussen et al. 2018; Jackson et al. 2018). Recanalization may affect to the long-term effectiveness of embolization and allow the recovery of normal tissue. The findings of the early recanalization of the embolized prostatic artery in the present study suggest it may also happen in human patients. Bilhim et al. (2016) reported that 20%–36% of patients still had moderate to severe LUTS after PAE, among which up to 80% of all were nonresponders who never substantially improved after PAE. However, how many nonresponders might have an early recanalization of the prostatic artery after PAE is unknown. The association of clinical failure with early prostatic recanalization needs to be addressed in clinical practice.

This study has several limitations. The sample size of animal was too small and lacked of comparison with other commonly used embolic agents in control group. The follow-up duration was just for 1 month without long-term observation. The absence of pathological evaluation over the recanalized vessels limited the strength of the study.

## Conclusions

The findings of the present study support that PAE with polyethylene glycol microspheres is a safe procedure, which is technically feasible and may induce local ischemia in the prostate, resulting in secondary glandular atrophy and shrinkage of prostate. More studies in large population of animals as well as with control groups are needed to further validate the therapeutic effectivity and safety. Imaging studies and pathological evaluation of the early recanalization of the target arteries after PAE remain to be further addressed in both laboratory investigation and clinical practice.

## Data Availability

The datasets generated and analyzed during the current study are available from the corresponding author on reasonable request.

## Abbreviations

BPH: Benign Prostatic Hyperplasia
MRI: Magnetic Resonance Imaging
NICE: National Institute of Health and Care Excellence
PAE: Prostatic Artery Embolization
PSA: Prostatic Specific Antigen
PV: Prostate Volume
TRUS: Transrectal ultrasonography
UK: United Kingdom
